# Surgical treatment of subependymal giant cell astrocytoma in patients with tuberous sclerosis complex—an institutional experience and results

**DOI:** 10.1007/s00381-025-06779-4

**Published:** 2025-03-04

**Authors:** Mia Tuft, Ylva Østby Berger, Pål Bache Marthinsen, Bernt Johan Due-Tønnessen, Radek Frič

**Affiliations:** 1 National Centre for Rare Epilepsy-related Disorders, Oslo University Hospital, P.O. Box 0495, N-0424 Oslo, Norway; 2 Institute of Psychology, University of Oslo, Oslo, Norway; 3 Deparment of Radiology and Nuclear Medicine, Oslo University Hospital-Rikshospitalet, Oslo, Norway; 4 Department of Neurosurgery, Oslo University Hospital-Rikshospitalet, Oslo, Norway; 5 Pediatric Neurosurgical Research Group, Faculty of Medicine, Institute of Clinical Medicine, University of Oslo, Oslo, Norway

**Keywords:** Tuberous sclerosis complex, Subependymal giant cell astrocytoma

## Abstract

**Objective:**

Subependymal giant cell astrocytomas (SEGA) are present in patients with tuberous sclerosis complex (TSC), occasionally requiring surgical removal. The study aimed to analyze the results from our series of children undergoing surgery for SEGA.

**Methods:**

We retrospectively identified children with TSC undergoing resection of SEGA at Oslo University Hospital between 1982 and 2016. Patient charts, radiological images, epilepsy, and neuropsychological reports were reviewed.

**Results:**

Out of 208 patients with TSC, 18 (9%) underwent resection of SEGA. Due to missing data, we could only analyze results from 14 surgeries in 11 children (median age 6 years, range 0–19; male/female ratio 2.7:1). The tumours were bilateral in four (36%) patients. The tumour diameter was a median of 19 mm (10–104 mm). The surgical approach was transcortical in eight (57%) and transcallosal in six surgeries (43%). Gross total resection was achieved in 12 (86%) of surgeries. There was no mortality or major morbidity related to surgery except for one case of chronic subdural hematoma, but out of two patients with ventriculoperitoneal shunts, one developed shunt infection, and both experienced shunt failures during the follow-up. During the follow-up (median 11 years, range 1–21), three patients (27%) underwent repeated surgery. We could not document any significant impact of the surgery on patients’ cognitive functioning or the grade of epilepsy.

**Conclusions:**

Resection of SEGA in children with TSC was associated with a low complication rate. We could not document any impact of surgery on patients’ cognitive functioning or grade of epilepsy. However, the neuropsychological data were limited in most cases. Neuropsychological assessment should be performed before the surgery and be a part of follow-up after surgery.

## Introduction

Subependymal giant cell astrocytomas (SEGA) are defined as a tumour typically located in the caudothalamic groove, with a size of more than 10 mm in any direction, or a subependymal lesion at any location that has shown serial growth on consecutive imaging regardless of size [1]. SEGA usually grow slowly, but their progression may ultimately lead to the occlusion of the foramen of Monro and the development of obstructive hydrocephalus. They occur in 10–25% of patients with tuberous sclerosis complex (TSC) [2, 3], but are extremely rare in the general population [4]. SEGA usually do not present in patients older than 20–25 years [3], but up to 57 years has been reported [2], with an average age of 10–11 years and a range of 3–20 years [5, 6].

Tuberous sclerosis complex (TSC), in which SEGA most often occur, is a genetic neurodevelopmental disorder that affects about 1 in 6.000–10.000 individuals and about 2 million people worldwide [7]. It is characterized by a tendency to develop tubers in different organs, most often skin, kidneys, brain, and heart. About 80–90% of individuals with TSC present with typical radiological changes in the brain and epilepsy [8].

Treatment of SEGA is surgical, with the removal of the tumour, or medical, with the use of mammalian target of rapamycin (mTOR) inhibitors [9–11]. Furthermore, apart from traditional open surgical techniques for the removal of SEGA using transcallosal or transcortical approaches as in our sample of patients, both the use of endoscopic surgical technique and laser interstitial thermal therapy (LITT) has been increasingly reported in recent years [12–16], although currently applicable only in tumours smaller than 3 cm.

Data on outcomes from surgical treatment of SEGA is essential for counselling the patients and their families, as well as health care workers. More information is needed in terms of cognitive outcomes that may have a significant impact on patients’ educational and occupational potential. Clinical experience with patients with TSC in Norway shows that these patients often are not adequately assessed regarding their cognitive level of functioning and neuropsychiatric issues. Such assessments are necessary to facilitate appropriate educational, occupational, and family care adjustments for a better level of functioning and quality of life. Similar observations have been reported from other countries [17]. International consensus recommendations for the identification and treatment of neuropsychiatric disorders in TSC state the importance of such assessment [17, 18]. A special checklist to ensure that each patient gets the right diagnosis and treatment is developed (The TAND Consortium) [19] and is available in Norwegian translation.

Epilepsy in TSC is mainly related to the genetic condition itself and tubers in the cerebral cortex. The grade of epilepsy before and after surgery of SEGA should be assessed with EEG and epilepsy diary. Only a few studies, including the present study and the previous French study [20], address this particular issue, as well as the neuropsychiatric aspects.

Surgical outcomes after surgical resection of SEGA in patients with TSC have been reported in previous studies including from two to 64 patients [5, 6, 16, 20–37]. These studies showed excellent outcomes with low morbidity and mortality in asymptomatic SEGA, whereas resection is associated with higher surgical morbidity and mortality when diagnosed at a later stage and with bigger tumours that affect surrounding brain structures such as the basal ganglia, hypothalamus, fornix, and genu of internal capsule [37]. Yet, there is a need for more knowledge, due to the small samples in all present studies.

Our aim was primarily to better understand the possible side effects of surgical resection of SEGA in the Norwegian patient population with TSC. We also wanted to investigate whether the surgical treatment of SEGA had an impact on patients’ cognitive and neuropsychiatric functioning and epilepsy.

## Material and methods

The study was approved by the regional ethical committee and Oslo University Hospital as a quality control study (reference number 2016/1187). We retrospectively analyzed medical records at Oslo University Hospital and The National Center for Rare Epilepsy-Related Disorders from 1982 to 2016 and identified all patients who underwent surgical resection of SEGA during this period.

Patient records were reviewed and the following information was retrieved: diagnosis of TSC; genetic test results; surgical notes; information about epilepsy, including EEG reports, seizure type, and frequency; pathology reports (to verify tumour classification); neuropsychology reports or other relevant information that could retrospectively describe the cognitive level of functioning; magnetic resonance imaging (MRI) or computed tomography (CT) scans. Surgical notes and pathology reports were reviewed by a neurosurgeon (R.F.), MRI and CT scans were reviewed by a neuroradiologist (P.B.M.), and information regarding epilepsy and cognitive functioning was reviewed by a neuropsychologist (M.T.).

Cognitive and behavioural functioning was assessed by analyzing all relevant information on the cognitive level of functioning, including test results from neuropsychological examinations (Wechsler Preschool and Primary Scale of Intelligence 3rd and 4th ed, The Beery-Buctencia Developmental Test of Visual_Motor Integration, 5th ed, BRIEF and PedsQL in the two presented cases), as well as information about diagnoses; reports on intellectual disability, ADHD, autism spectrum disorder, and behaviour; pedagogical reports; and other relevant clinical descriptions.

Epilepsy was classified into five grades: 0 (no epilepsy), 1 (no current epilepsy, no drugs), 2 (seizures controlled with drugs), 3 (weekly seizures), and 4 (daily seizures). We also noted whether there were focal seizures, Lennox Gastaut syndrome, or non-epileptic seizures.

## Results

### Patient sample

We identified 208 patients with TSC: 205 from the register at The National Center for Rare Epilepsy-Related Disorders and three from a journal search on the diagnosis (Q85.1 Tuberous Sclerosis Complex) at Oslo University Hospital. Out of these, we found 34 (17%) patients harbouring SEGA, of whom 18 patients (9%) underwent surgical resection.

Six (33%) of the 18 patients had a mutation of TSC1 and eight (44%) of TSC2, while four (22%) were not genetically specified. Pathology records confirming SEGA were available in only 11 patients; therefore, the remaining cases were excluded from further analysis. The male/female ratio was 2.7:1; the median age at the time of the first surgery was 6 years (range 0–19) (Table 1).

**Table 1:** Characteristics of the patient sample in the present study

Patient	Sex	Age^1^	Surgery	epi	Gene	Location	Side	Size (max, mm)	HC	Surgical approach	Resection grade	Histology verified	Follow-up (years)	Recurrence	Complications
1	M	19	2001	Yes	?	FH	R	15	No	Transcortical	GTR	Yes	12	No	None
2	M	10	2001	Yes	TSC1	FH	R	34	Yes	Transcortical	GTR	Yes	11^†^	No	Shunt infection, Shunt failure
3	M	5	2002	Yes	TSC1	FH	L	25	No	Transcortical	GTR	Yes	21	No	None
4	M	0	2006	Yes	TSC2	FT	R	57	Yes	Transcortical	STR	Yes	10^†^	Yes	Shunt failure
		3	2009			FT	R	104	Yes	Transcortical	STR	Yes	7	No	None
5	F	6	2008	Yes	TSC2	FH	Bilat	12L, 24R	Yes	Transcortical	GTR	Yes	15	No	None
6	F	5	2008	Yes	TSC1	FH	L	20	No	Transcortical	GTR	Yes	12	Yes	None
		16	2019	No		FH	L	15	No	Transcortical	GTR	Yes	1	No	None
7	F	9	2009	Yes	TSC1	FH	L	28	No	Transcallosal	GTR	Yes	1	No	None
8	M	6	2012	Yes	TSC1	FH	Bilat	16L, 10R	No	Transcallosal	GTR	Yes	11	Yes	None
9	M	6	2013	Yes	TSC2	FH	Bilat	20L, 17R	No	Transcallosal	GTR	Yes	9	No	None
10	M	10	2016	Yes	TSC2	FH	Bilat	19L, 13R	Yes	Transcallosal	GTR	Yes	7	Yes	Chronic SDH
		16	2022	Yes		FH	Bilat	22L, 14R	Yes	Transcallosal	GTR	Yes	1	No	None
11	M	8	2016	Tes	TSC2	FH	L	19	Yes	Transcallosal	GTR	Yes	7	No	None

^1^At the time of the first surgery. *GTR* gross total resection, *FH* frontal horn, *FT* frontotemporal, *HC* hydrocephalus, *L* left, *R* right, *SDH* subdural hematoma, *STR* subtotal resection. ^†^Deceased

### Radiology

**Fig. 1:**
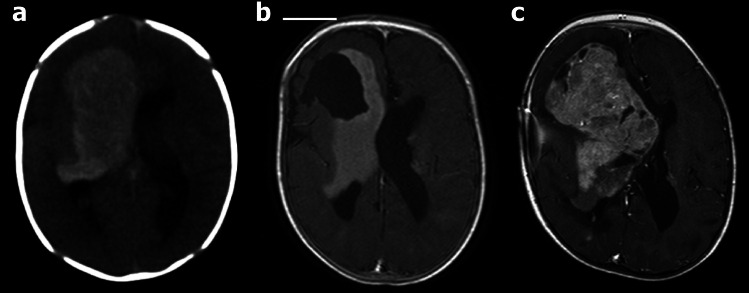
A case of a large SEGA in the right cerebral hemisphere, detected at birth (patient no. 4 in Tables 1 and 2). **a** Only CT was performed after birth and before the first emergency surgery when the tumour was only partially removed to achieve decompression and obtain material for histological diagnosis. **b** Axial MRI T1-weighted post-contrast image (T1 CE) acquired after the first surgery and biopsy showed partial removal of the tumour. **c** MRI scan obtained 3 years later showed tumour growth, resulting in the second surgical resection

Most of the SEGAs were in the typical location in the frontal ventricular horns, except for one case of a large frontotemporal (hemispheric) tumour (Fig. 1). The lesions were located on the right side in three (27%), on the left side in four (36%), and bilateral in four (36%) patients. The tumour diameter was median 19 mm (range 10–104 mm). Five patients (46%) had hydrocephalus (Table 1), although radiologically only mild in three of these cases.

### Surgery

Fourteen tumour resections were performed in 11 patients. The surgical approach was transcortical in eight (57%) and transcallosal in six cases (43%). Gross total resection (GTR) was achieved in 12 out of 14 (86%) of surgeries. Two patients (18%) received a ventriculoperitoneal shunt (VPS), in one case before the surgical resection of SEGA. We experienced no mortality or major morbidity related to surgery of SEGA other than a chronic subdural hematoma in one patient (7% of all 14 resections), but one patient with VPS (9%) developed shunt infection and both patients with VPS experienced shunt failures during the follow-up.

During the follow-up of the median 11 years (range 1–21) following the first surgery, three patients (27%) underwent repeated surgery for SEGA due to radiologically documented recurrences. Two patients died from complications to their diagnosis of TSC, but not related to surgery.

### Epilepsy

All patients had epileptic seizures before surgery, and the grade of epilepsy remained unchanged after surgery (Table 2).

**Table 2:** Functional characteristics (epilepsy, cognitive skills, autism, and behaviour) of the patients included in the study

Patient	Epilepsy grade		Cognitive skills^1^	ASD and autistic traits^2^	Behaviour difficulties
	Before surgery	After surgery			
1	4	4	Moderate ID	Yes	Not verified
2	4	4	Deep ID	Yes	Yes
3	2	2	Normal	No	No
4	4 (LGS)	4 (LGS)	Deep ID	Yes	No
5	4	4	Unspecified ID	Yes	Yes
6	0–1^3^	0^3^	Normal	No	No
7	2	2	IQ scores lower normal	No	No
8	4 (FS)	4 (FS)	Moderate-severe ID	Traits	Yes
9	4 (FS)	4 (FS)	Reduced (equally to 4–5 ys at 6 ys of age), ID (unspecified)	Traits	Yes + ADHD
10	4 (LGS)	4 (LGS)	Severe-deep ID (IQ < 20)	Yes	Yes
11	2	2	Mild ID	Traits	Described behavioural problems

Epilepsy grade: Grade 0 = no epilepsy; Grade 1 = no current epilepsy, no drugs; Grade 2 = seizures controlled with drugs; Grade 3 = weekly seizures; Grade 4 = daily seizures. *FS* focal seizures, *ID* intellectual disability, *LGS* Lennox Gastaut syndrome, *na* not available. Before and after surgery = the last EEG before surgery and the EEGs the following years after surgery

^1^Mild ID, IQ 50–69; moderate ID, IQ 35–49; severe ID, IQ 20–34; profound ID, IQ < 20; unspecified ID, below IQ 70. Normal = normal IQ results or no special education or normal full-time work as an adult

^2^ASD (autism spectrum disorder) refers to the diagnosis given by a clinician, and autistic traits refer to clinical signs of autism spectrum disorder

^3^Used medication prophylactically, but did not have epileptic seizures; stopped taking medication 6 months after surgery and remained seizure-free

### Neuropsychology

Neuropsychological assessments were scarcely described in the records of most patients, but in the majority of cases, it was possible to gather broad estimates based on journal descriptions and cognitive disability diagnoses, as seen in Table 2. Based on these descriptions of cognitive and behavioural functioning, no patients experienced any noticeable changes after surgery.

Only two patients (nos. 6 and 7 in Table 2) were neuropsychologically assessed both before and after surgery and may be described briefly here:

#### A case report (patient no. 10)

The testing of patient no. 10 (Tables 1 and 2), a 10-year-old boy with autism and severe to deep intellectual disability, was performed the day before the SEGA surgery and adjusted to the individual cognitive level of functioning. As the hospital had still not established routines for testing this group of patients before and after surgery, the conditions for testing were suboptimal. The boy was easily distracted, and appeared motorically restless, but cooperated when rewarded with iPad puzzles.

The investigation was based on the Bayley Scales of Infant and Toddler Development, Third Edition (cognitive and language scale) [38], Behaviour Rating Inventory of Executive Function (BRIEF), and PEDS QL Life quality in children version 4.0 [39], translated into Norwegian. Additional information was given from his mother and father, who were there during the first test sessions.

The postsurgical assessment was performed by the same neuropsychologist and took place at the boy’s school, with his special education teacher present throughout the session. The Bayley Scales (cognitive and language scale) was used for the post-surgery assessment, as well as observation of activities at his school teaching session with his special education teacher, and a clinical interview with the parents. His special education teacher supplied information about his level of words, and which specific words he knew and could pronounce.

The boy had a slightly better performance when tested 1 year after surgery. Before surgery, his test results were equivalent with children of 19–20 months on the cognitive scale and equivalent with 12–14 months old children on the scale for expressive communication. One year later, at the age of 11, his results had increased slightly, to the average of 21–22 months and 18–19 months on the same subscales, respectively. His mother reported that he had learned some more words than a year ago. The questionnaires BRIEF and PedsQL before surgery verified a need for comprehensive care. As expected, the answers verified severe problems with impulse control, attention and shifting attention from one activity to another, emotional control, social skills and communication, working memory, planning, and organizing. The same questionnaires were not filled out after surgery, but the clinical interview with the mother revealed a slight general improvement a year after surgery. He was more often in a good mood than he was before surgery, and he had shown a slight cognitive improvement and no stagnation in any area. He had learned some new skills, and the mother reported that a normal day in general was better than before surgery, in terms of life satisfaction. The information was supported by his special education teacher.

#### A case report (patient no. 11)

The patient, a 7.5-year-old boy, was assessed by the same psychologist (M.T.) the day before the surgery for SEGA and 1 year after surgery. Wechsler Preschool and Primary Scale of Intelligence, 3rd and 4th editition (WPPSI-III/IV) [40, 41]; the Beery-Buctencia Developmental Test of Visual-Motor Integration, 5th edition (Beery VMI) [42]; and Grooved Pegboard Test [43] were used in the first assessment. The patient’s mother filled out the questionnaires BRIEF and Paediatric Quality of Life Inventory (PedsQL). At the second assessment, the same assessment tools and questionnaires were administered, except that the third version of WPPSI had to be used, as the 4th version was unavailable at the time of testing. In both instances, test sessions were cut short because of a lack of compliance and/or endurance or because of difficulty understanding instructions. Additional anamnestic information was provided by the patient’s mother and teacher.

Before surgery, at the age of 7.5 years, the test Vocabulary from WPPSI-IV [44] scores were equivalent to the mean for 2.5-year-old children, and the test Picture Naming from the same test battery gave scores equal to mean scores for a 4-year-old child. He did not understand the part test Block Design. At a test of fine motor skills and speed (Grooved Pegboard), the scores were only slightly below mean scores for his age and there were no significant differences between his left and right hand. On a drawing task, he managed to copy simple geometrical figures (circles, simple lines, triangles, squares), but not any advanced figures. His results were equivalent to 5-year-old children. Results from the BRIEF questionnaire revealed problems within several areas: behavioural inhibition, transitions between activities, working memory, planning, organizing, and self-monitoring in social relations. Answers from the questionnaire PedsQL revealed some difficulties within social functioning and school functioning.

After surgery, at the age of 8 years, scores were equivalent to the mean scores for 4-year-olds on the subtests Information and Vocabulary and scores equivalent to mean scores for 3-year-olds on the subtest Block Design. It was not possible to calculate a full IQ score, because he lost patience and motivation in the test situation. On a test for fine motoric speed (Grooved Pegboard) [43], his scores were somewhat below the mean scores for his age, but like before the surgery, he mastered this skillfully, and there were no differences between his left and right hands. On a drawing task, copying geometrical figures, his results were equivalent to 6-year-old children.

The BRIEF questionnaire indicated that he had improved in several areas. He still had problems with transitions and had problems with self-monitoring (but it was a lot better than at the last assessment), planning, organizing, and working memory. However, his inhibition skills were improved, as were his behavioural regulation and social skills. In general, the mean scores were higher than before surgery.

Results from the questionnaire PedsQL were almost the same as the previous assessment, but there was an improvement in social function and school functioning.

## Discussion

The rationale for the treatment of SEGA is primarily to alleviate the mass effect and secondary hydrocephalus caused by the tumour. In the most recent literature review of 18 studies published between 1980 and 2021 counting a total of 263 patients [36], the surgical morbidity was 4.9% and morbidity 33.6%, while ventriculoperitoneal was needed in 30.8% of cases. Although the comparison is difficult due to the low number of cases in the present series, our results are more in line with own data from the same study, showing very few complications and negligible impact of surgical removal of SEGA on the grade of epilepsy in the identical number of patients [36].

While surgical treatment of symptomatic or growing SEGA remains a valid treatment strategy, the risk of significant neurological morbidity reported in older publications (5–50%) is still considered its main limitation [15, 31, 36]. The risk of complications appears particularly high in large SEGA [37], in which the use of alternative treatment methods—such as laser interstitial thermal therapy (LITT) and mTOR inhibitors—is limited. It is therefore important to regularly screen TSC patients for SEGA and initiate early treatment when necessary.

Importantly, the present case series includes also patients treated already in the late 1990s and early 2000s, before the implementation of the new recommendations regarding the management of SEGA including the use of mTOR inhibitors [7]. Medical treatment with mTOR inhibitors was not available as an alternative to surgery for SEGA until around 2010 [45]. The advantageous effect of mTOR inhibitors is the shrinking of SEGA when tumours are large and infiltrate surrounding brain tissue. It can be used as neoadjuvant treatment before surgery [46] and on a long-term basis. A study showed that more than 60% of patients receiving treatment for ≥ 5 years exhibited a clinically relevant (≥ 30%) reduction of their SEGA [45]. In a Norwegian-Danish study, five patients were treated with everolimus for the indication of SEGA, achieving volume reduction in three of them; this study also stressed the importance of frequent and close evaluation of side effects [47]. Side effects of mTOR inhibitors used over a long time are still unknown, because of still limited long-term experience with these drugs. Moreover, there may be differences in side effects seen in children and adults [47, 48]. A major disadvantage is the recurrence of SEGA growth when the medication is discontinued [49].

Besides the medical treatment of SEGA, mTOR inhibitors are also used for the treatment of other manifestations of TSC [50].

We gathered data from 11 consecutive patients treated during the last two decades. Another seven patients were treated during the previous two decades (before 2000); here, the data on pathohistological verification were lacking, and these cases were therefore excluded from further analysis. However, it is still reasonable to assume that they also had SEGA, as SEGA is almost exclusively related to TSC and these were growing tumours. The available clinical patterns from these excluded cases were very similar to those presented in Tables 1 and 2: the semiology of epilepsy was similar and there were no major changes in the grade of epilepsy after surgery (except that epilepsy increased in one case and decreased in another) and no cognitive or neuropsychiatric changes related to surgery, either.

The low number of cases available from our tertiary high-volume institution reflects the rarity of SEGA, which presents in only 10–25% of patients with TSC [2, 3], and is in line with numbers presented in most other surgical series [5, 13, 16, 20, 22, 24, 29, 32, 33, 36]. Studies from larger samples of patients are rare [31, 35, 37], particularly after the introduction of medical treatment with mTOR inhibitors [51]. In Norway, we found that 17% of patients with TSC had SEGA, according to patient records compared with a national registry.

The grade of epilepsy remained unchanged in all patients. In general, epilepsy is present in about 85% of all patients with TSC, and up to 75% have seizures that are difficult to treat [52, 53]. Surgical treatment of SEGA, regardless of the surgical approach, does not seem to worsen epilepsy. A literature review reported that only three patients had new seizures after surgery, some patients improved, and the rest remained unchanged [36]. In the present study, we used a grading system from 0 to 4 for better comparison with the study of Fohlen and colleagues [20], since epilepsy outcomes are scarcely described in the surgical literature.

One important finding of the present retrospective study was the lack of systematic, standardized examination of cognitive functions in our historical series of patients with SEGA. The impression that also cognitive and behavioural functioning was mostly unaffected by the surgery in our series is therefore based on a certain confidence in the clinical information stated in the medical records. Ideally, this information should have come from dedicated clinical assessments. For the patients, their families, and medical personnel, information about cognitive and behavioural functioning should be an integral part of a preoperative workup and postoperative follow-up. According to a national tumour follow-up program for children in Norway, all children undergoing resection of a brain tumour are to be assessed by a neuropsychologist before and after surgery [54]. It may be suspected that patients with SEGA associated with TSC, which is a rare diagnosis, were not primarily regarded as brain tumour patients and therefore slipped from this routine. Another explanation could be that many of the patients have autism and intellectual disability, and their neuropsychological assessment was not considered feasible by clinicians. TSC is a diagnosis with a considerable range in terms of level of functioning [17]: if the patients with SEGA have a normal intellectual capacity and no identifiable neuropsychiatric symptoms, they can be tested by standard test batteries for their age, but most children with TSC have one or more neuropsychiatric problems, including intellectual disabilities, autism, and ADHD. The neuropsychological assessments therefore require individual adjustment. Although SEGA presents mostly in children, there are a few exceptions and individual adjustment may be needed also in adult patients. The TAND consortium (tandconsortium.org) with dedicated checklists can be used as a tool for neuropsychiatric screening for all patients with TSC, and further assessments should be carried out according to the screening results [19].

### Limitations

The data in this study are retrieved retrospectively and based mostly on available patient records with clinical descriptions of cognitive functioning to epilepsy and reported surgical complications. The number of patients is small and therefore does not allow for any recommendations or relevant statistical analysis.

## Conclusions

Surgical resection of SEGA in children with TSC was associated with a low complication rate. Recurrences were mostly related to subtotal resection. We could not document any impact of surgery on patients’ cognitive function or grade of epilepsy. However, the main finding was that neuropsychological data were limited in the vast majority of the cases. Neuropsychological assessment should be performed before the surgery and be a part of follow-up after surgery.

## Data Availability

No datasets were generated or analysed during the current study.

## References

[1] Roth J, Roach ES, Bartels U, Jóźwiak S, Koenig MK, Weiner HL, Franz DN, Wang HZ (2013) Subependymal giant cell astrocytoma: diagnosis, screening, and treatment. Recommendations from the International Tuberous Sclerosis Complex Consensus Conference 2012. Pediatr Neurolol 49:439–444 10.1016/j.pediatrneurol.2013.08.017 24138953

[2] Jansen AC, Belousova E, Benedik MP, Carter T, Cottin V, Curatolo P, D’Amato L, Beaure d’Augères G, de Vries PJ, Ferreira JC, Feucht M, Fladrowski C, Hertzberg C, Jozwiak S, Lawson JA, Macaya A, Marques R, Nabbout R, O’Callaghan F, Qin J, Sander V, Sauter M, Shah S, Takahashi Y, Touraine R, Youroukos S, Zonnenberg B, Kingswood JC (2019) Newly diagnosed and growing subependymal giant cell astrocytoma in adults with tuberous sclerosis complex: results from the International TOSCA study. Front Neurol 10:821 31428037 10.3389/fneur.2019.00821 PMC6688052

[3] Adriaensen ME, Schaefer-Prokop CM, Stijnen T, Duyndam DA, Zonnenberg BA, Prokop M (2009) Prevalence of subependymal giant cell tumors in patients with tuberous sclerosis and a review of the literature. Eur J Neurol 16:691–696 19236458 10.1111/j.1468-1331.2009.02567.x

[4] Decq P, Le Guerinel C, Sol JC, Brugieres P, Djindjian M, Nguyen JP (2001) Chiari I malformation: a rare cause of noncommunicating hydrocephalus treated by third ventriculostomy. J Neurosurg 95:783–790 11702868 10.3171/jns.2001.95.5.0783

[5] Goh S, Butler W, Thiele EA (2004) Subependymal giant cell tumors in tuberous sclerosis complex. Neurology 63:1457–1461 15505165 10.1212/01.wnl.0000142039.14522.1a

[6] Cuccia V, Zuccaro G, Sosa F, Monges J, Lubienieky F, Taratuto AL (2003) Subependymal giant cell astrocytoma in children with tuberous sclerosis. Childs Nerv Syst 19:232–243 12715190 10.1007/s00381-002-0700-2

[7] Northrup H, Aronow ME, Bebin EM, Bissler J, Darling TN, de Vries PJ, Frost MD, Fuchs Z, Gosnell ES, Gupta N, Jansen AC, Jóźwiak S, Kingswood JC, Knilans TK, McCormack FX, Pounders A, Roberds SL, Rodriguez-Buritica DF, Roth J, Sampson JR, Sparagana S, Thiele EA, Weiner HL, Wheless JW, Towbin AJ, Krueger DA (2021) Updated international tuberous sclerosis complex diagnostic criteria and surveillance and management recommendations. Pediatr Neurol 123:50–66 34399110 10.1016/j.pediatrneurol.2021.07.011

[8] Henske EP, Jóźwiak S, Kingswood JC, Sampson JR, Thiele EA (2016) Tuberous sclerosis complex. Nat Rev Dis Primers 2:16035 27226234 10.1038/nrdp.2016.35

[9] Tomoto K, Fujimoto A, Inenaga C, Okanishi T, Imai S, Ogai M, Fukunaga A, Nakamura H, Sato K, Obana A, Masui T, Arai Y, Enoki H (2021) Experience using mTOR inhibitors for subependymal giant cell astrocytoma in tuberous sclerosis complex at a single facility. BMC Neurol 21:139 33784976 10.1186/s12883-021-02160-5 PMC8011204

[10] Cappellano AM, Senerchia AA, Adolfo F, Paiva PM, Pinho R, Covic A, Cavalheiro S, Saba N (2013) Successful everolimus therapy for SEGA in pediatric patients with tuberous sclerosis complex. Childs Nerv Syst 29:2301–2305 23743818 10.1007/s00381-013-2170-0

[11] Franz DN, Belousova E, Sparagana S, Bebin EM, Frost M, Kuperman R, Witt O, Kohrman MH, Flamini JR, Wu JY, Curatolo P, de Vries PJ, Whittemore VH, Thiele EA, Ford JP, Shah G, Cauwel H, Lebwohl D, Sahmoud T, Jozwiak S (2013) Efficacy and safety of everolimus for subependymal giant cell astrocytomas associated with tuberous sclerosis complex (EXIST-1): a multicentre, randomised, placebo-controlled phase 3 trial. Lancet 381:125–132 23158522 10.1016/S0140-6736(12)61134-9

[12] Aum DJ, Reynolds RA, McEvoy SD, Wong M, Roland JL, Smyth MD (2024) Laser interstitial thermal therapy compared with open resection for treating subependymal giant cell astrocytoma. J Neurosurg Pediatr 33:95–104 37922551 10.3171/2023.8.PEDS23370

[13] Boop S, Bonda D, Randle S, Leary S, Vitanza N, Crotty E, Novotny E, Friedman S, Ellenbogen RG, Durfy S, Goldstein H, Ojemann JG, Hauptman JS (2023) A comparison of clinical outcomes for subependymal giant cell astrocytomas treated with laser interstitial thermal therapy, open surgical resection, and mTOR inhibitors. Pediatr Neurosurg 58:150–159 37232001 10.1159/000531210

[14] Desai VR, Jenson AV, Hoverson E, Desai RM, Boghani Z, Lee MR (2020) Stereotactic laser ablation for subependymal giant cell astrocytomas: personal experience and review of the literature. Childs Nerv Syst 36:2685–2691 32468241 10.1007/s00381-020-04638-y

[15] Frassanito P, Noya C, Tamburrini G (2020) Current trends in the management of subependymal giant cell astrocytomas in tuberous sclerosis. Childs Nerv Syst 36:2527–2536 32978642 10.1007/s00381-020-04889-9

[16] Cai R, Di X (2010) Combined intra- and extra-endoscopic techniques for aggressive resection of subependymal giant cell astrocytomas. World Neurosurg 73:713–718 20934162 10.1016/j.wneu.2010.02.068

[17] de Vries PJ, Heunis TM, Vanclooster S, Chambers N, Bissell S, Byars AW, Flinn J, Gipson TT, van Eeghen AM, Waltereit R, Capal JK, Cukier S, Davis PE, Smith C, Kingswood JC, Schoeters E, Srivastava S, Takei M, Gardner-Lubbe S, Kumm AJ, Krueger DA, Sahin M, De Waele L, Jansen AC (2023) International consensus recommendations for the identification and treatment of tuberous sclerosis complex-associated neuropsychiatric disorders (TAND). J Neurodev Disord 15(1):32 37710171 10.1186/s11689-023-09500-1 PMC10503032

[18] de Vries P, Humphrey A, McCartney D, Prather P, Bolton P, Hunt A (2005) Consensus clinical guidelines for the assessment of cognitive and behavioural problems in Tuberous Sclerosis. Eur Child Adolesc Psychiatry 14:183–190 15981129 10.1007/s00787-005-0443-1

[19] The TAND Consortium CHECKLISTS - TANDem (tandconsortium.org).

[20] Fohlen M, Ferrand-Sorbets S, Delalande O, Dorfmüller G (2018) Surgery for subependymal giant cell astrocytomas in children with tuberous sclerosis complex. Childs Nerv Syst 34:1511–1519 29766265 10.1007/s00381-018-3826-6

[21] Sinson G, Sutton LN, Yachnis AT, Duhaime AC, Schut L (1994) Subependymal giant cell astrocytomas in children. Pediatr Neurosurg 20:233–239 8043461 10.1159/000120796

[22] Roszkowski M, Drabik K, Barszcz S, Jozwiak S (1995) Surgical treatment of intraventricular tumors associated with tuberous sclerosis. Childs Nerv Syst 11:335–339 7671268 10.1007/BF00301665

[23] Di Rocco C, Iannelli A, Marchese E (1995) On the treatment of subependymal giant cell astrocytomas and associated hydrocephalus in tuberous sclerosis. Pediatr Neurosurg 23:115–121 8751291 10.1159/000120947

[24] Turgut M, Akalan N, Ozgen T, Ruacan S, Erbengi A (1996) Subependymal giant cell astrocytoma associated with tuberous sclerosis: diagnostic and surgical characteristics of five cases with unusual features. Clin Neurol Neurosurg 98:217–221 8884092 10.1016/0303-8467(96)00028-5

[25] Sharma MC, Ralte AM, Gaekwad S, Santosh V, Shankar SK, Sarkar C (2004) Subependymal giant cell astrocytoma–a clinicopathological study of 23 cases with special emphasis on histogenesis. Pathol Oncol Res 10:219–224 15619643 10.1007/BF03033764

[26] Kumar R, Singh V (2004) Subependymal giant cell astrocytoma: a report of five cases. Neurosurg Rev 27:274–280 15309659 10.1007/s10143-004-0339-4

[27] de Ribaupierre S, Dorfmüller G, Bulteau C, Fohlen M, Pinard JM, Chiron C, Delalande O (2007) Subependymal giant-cell astrocytomas in pediatric tuberous sclerosis disease: when should we operate? Neurosurgery 60:83–89 (discussion 89-90) 17228255 10.1227/01.NEU.0000249216.19591.5D

[28] Pascual-Castroviejo I (2011) Neurosurgical treatment of tuberous sclerosis complex lesions. Childs Nerv Syst 27:1211–1219 21607641 10.1007/s00381-011-1488-8

[29] Jiang T, Jia G, Ma Z, Luo S, Zhang Y (2011) The diagnosis and treatment of subependymal giant cell astrocytoma combined with tuberous sclerosis. Childs Nerv Syst 27:55–62 20422196 10.1007/s00381-010-1159-1

[30] Ekici MA, Kumandas S, Per H, Ekici A, Tucer B, Gumus H, Canoz O, Kurtsoy A (2011) Surgical timing of the subependymal giant cell astrocytoma (SEGA) with the patients of tuberous sclerosis complex. Turk Neurosurg 21:315–324 21845566 10.5137/1019-5149.JTN.4169-11.0

[31] Sun P, Kohrman M, Liu J, Guo A, Rogerio J, Krueger D (2012) Outcomes of resecting subependymal giant cell astrocytoma (SEGA) among patients with SEGA-related tuberous sclerosis complex: a national claims database analysis. Curr Med Res Opin 28:657–663 22375958 10.1185/03007995.2012.658907

[32] Amin S, Carter M, Edwards RJ, Pople I, Aquilina K, Merrifield J, Osborne JP, O’Callaghan FJ (2013) The outcome of surgical management of subependymal giant cell astrocytoma in tuberous sclerosis complex. Eur J Pediatr Neurol 17:36–44 10.1016/j.ejpn.2012.10.005 23183057

[33] Harter DH, Bassani L, Rodgers SD, Roth J, Devinsky O, Carlson C, Wisoff JH, Weiner HL (2014) A management strategy for intraventricular subependymal giant cell astrocytomas in tuberous sclerosis complex. J Neurosurg Pediatr 13:21–28 24180681 10.3171/2013.9.PEDS13193

[34] Katz JS, Frankel H, Ma T, Zagzag D, Liechty B, Zeev BB, Tzadok M, Devinsky O, Weiner HL, Roth J (2017) Unique findings of subependymal giant cell astrocytoma within cortical tubers in patients with tuberous sclerosis complex: a histopathological evaluation. Childs Nerv Syst 33:601–607 28074282 10.1007/s00381-017-3335-z

[35] Giordano F, Moscheo C, Lenge M, Biagiotti R, Mari F, Sardi I, Buccoliero AM, Mongardi L, Aronica E, Guerrini R, Genitori L (2020) Neurosurgical treatment of subependymal giant cell astrocytomas in tuberous sclerosis complex: a series of 44 surgical procedures in 31 patients. Childs Nerv Syst 36:951–960 31853898 10.1007/s00381-019-04449-w

[36] Danassegarane G, Tinois J, Sahler Y, Aouaissia S, Riffaud L (2022) Subependymal giant-cell astrocytoma: a surgical review in the modern era of mTOR inhibitors. Neurochirurgie 68:627–636 35907444 10.1016/j.neuchi.2022.07.003

[37] Kotulska K, Borkowska J, Roszkowski M, Mandera M, Daszkiewicz P, Drabik K, Jurkiewicz E, Larysz-Brysz M, Nowak K, Grajkowska W, Domańska-Pakieła D, Jóźwiak S (2014) Surgical treatment of subependymal giant cell astrocytoma in tuberous sclerosis complex patients. Pediatr Neurol 50:307–312 24507694 10.1016/j.pediatrneurol.2013.12.004

[38] Gioia GA, Isquith PK, Guy SC, Kenworthy L (2000) TEST REVIEW Behavior rating inventory of executive function. Child Neuropsychol 6:235–238 11419452 10.1076/chin.6.3.235.3152

[39] Varni JW, Seid M, Rode CA (1999) The PedsQL: measurement model for the pediatric quality of life inventory. Med Care 37:126–139 10024117 10.1097/00005650-199902000-00003

[40] D W, Intelligence WPaPSo (2010) re year 2:6–3:11 08 Trykk AB S (2010) (3rd ed.) Year 2:(6–3):11. 08 Trykk AB, Stockholm

[41] Weschler D (2009) Weschler D III, WISC Weschler intelligence scale for children. Norwegian version, 4th ed, Katarina Tryck AB, Stockholm

[42] Beery KE BN, Beery VMI (2006) The Beery-Buktencia development test of visual-motor integration (6th ed.), Pearson Clinical Assessment, San Antonio

[43] Merker B, Podell K (2011) Grooved pegboard test. In: Kreutzer JS, DeLuca J, Caplan B (eds) Encyclopedia of clinical neuropsychology. Springer, New York, NY

[44] D W (2012) Wechsler preschool and primary scale of intelligence – fourth edition WPPSI-IV. Pearon, 2012 10.1037/spq0000038 24188289

[45] Franz DN, Agricola K, Mays M, Tudor C, Care MM, Holland-Bouley K, Berkowitz N, Miao S, Peyrard S, Krueger DA (2015) Everolimus for subependymal giant cell astrocytoma: 5-year final analysis. Ann Neurol 78:929–938 26381530 10.1002/ana.24523 PMC5063160

[46] Jiang T, Du J, Raynald WJ, Li C (2017) Presurgical administration of mTOR inhibitors in patients with large subependymal giant cell astrocytoma associated with tuberous sclerosis complex. World Neurosurg 107:1053.e1051-1053.e1056 10.1016/j.wneu.2017.08.122 28866062

[47] Cockerell I, Christensen J, Hoei-Hansen CE, Holst L, Grenaa Frederiksen M, Issa-Epe AI, Nedregaard B, Solhoff R, Heimdal K, Johannessen Landmark C, Lund C, Nærland T (2023) Effectiveness and safety of everolimus treatment in patients with tuberous sclerosis complex in real-world clinical practice. Orphanet J Rare Dis 18:377 38042867 10.1186/s13023-023-02982-1 PMC10693167

[48] Curatolo P, Franz DN, Lawson JA, Yapici Z, Ikeda H, Polster T, Nabbout R, de Vries PJ, Dlugos DJ, Fan J, Ridolfi A, Pelov D, Voi M, French JA (2018) Adjunctive everolimus for children and adolescents with treatment-refractory seizures associated with tuberous sclerosis complex: post-hoc analysis of the phase 3 EXIST-3 trial. Lancet Child Adolesc Health 2:495–504 30169322 10.1016/S2352-4642(18)30099-3

[49] Campen CJ, Porter BE (2011) Subependymal giant cell astrocytoma (SEGA) Treatment update. Curr Treat Options Neurol 13:380–385 21465222 10.1007/s11940-011-0123-z PMC3130084

[50] Curatolo P, Specchio N, Aronica E (2022) Advances in the genetics and neuropathology of tuberous sclerosis complex: edging closer to targeted therapy. Lancet Neurol 21:843–856 35963265 10.1016/S1474-4422(22)00213-7

[51] Ryoo JS, Khalid SI, Chaker AN, Behbahani M, Nunna RS, Mehta AI (2021) Trends in survival and treatment of SEGA: national cancer database analysis. Neurooncol Pract 8:98–105 33664974 10.1093/nop/npaa060 PMC7906259

[52] Curatolo P, Moavero R, de Vries PJ (2015) Neurological and neuropsychiatric aspects of tuberous sclerosis complex. Lancet Neurol 14:733–745 26067126 10.1016/S1474-4422(15)00069-1

[53] Curatolo P, Nabbout R, Lagae L, Aronica E, Ferreira JC, Feucht M, Hertzberg C, Jansen AC, Jansen F, Kotulska K, Moavero R, O’Callaghan F, Papavasiliou A, Tzadok M, Jóźwiak S (2018) Management of epilepsy associated with tuberous sclerosis complex: updated clinical recommendations. Eur J Pediatr Neurol 22:738–748 10.1016/j.ejpn.2018.05.006 29880258

[54] Guide from The Directory of Health, Norway: Nevropsykologisk oppfølging etter behandling for hjernesvulst - Helsedirektoratet

